# Gait Optimization Method for Humanoid Robots Based on Parallel Comprehensive Learning Particle Swarm Optimizer Algorithm

**DOI:** 10.3389/fnbot.2020.600885

**Published:** 2021-01-15

**Authors:** Chongben Tao, Jie Xue, Zufeng Zhang, Feng Cao, Chunguang Li, Hanwen Gao

**Affiliations:** ^1^School of Electronic and Information Engineering, Suzhou University of Science and Technology, Suzhou, China; ^2^Suzhou Automobile Research Institute, Tsinghua University, Suzhou, China; ^3^Department of Automation, Tsinghua University, Beijing, China; ^4^Wuhan Electronic Information Institute, Hubei, China; ^5^School of Computer and Information Technology, Shanxi University, Taiyuan, China; ^6^School of Computer Information and Engineering, Changzhou Institute of Technology, Changzhou, China

**Keywords:** RoboCup3D, humanoid robot, PCLPSO, parallel distributed, layered learning

## Abstract

To improve the fast and stable walking ability of a humanoid robot, this paper proposes a gait optimization method based on a parallel comprehensive learning particle swarm optimizer (PCLPSO). Firstly, the key parameters affecting the walking gait of the humanoid robot are selected based on the natural zero-moment point trajectory planning method. Secondly, by changing the slave group structure of the PCLPSO algorithm, the gait training task is decomposed, and a parallel distributed multi-robot gait training environment based on RoboCup3D is built to automatically optimize the speed and stability of bipedal robot walking. Finally, a layered learning approach is used to optimize the turning ability of the humanoid robot. The experimental results show that the PCLPSO algorithm achieves a quickly optimal solution, and the humanoid robot optimized possesses a fast and steady gait and flexible steering ability.

## Introduction

Gait planning is a research hotspot for humanoid robots, and it provides some technical support for humanoid robots walking like humans. The methods of gait planning can be broadly divided into three categories: human walking parameter-based methods (Baoping et al., 2015; Hereid et al., 2018), humanoid walking model-based methods (Sato et al., 2010; Winkler et al., 2018), and intelligent algorithm-based methods (Huan and Anh, 2015; Elhosseini et al., 2019). The method based on human walking parameters makes the gait of humanoid robots more similar to the way humans walk, but it costs a lot of time to find suitable gait parameters from human walking data and apply them to humanoid robots. Paparisabet et al. (2019) proposed a similar function for human-like motion, formulated kinematic constraints for humanoid robots in contact with the ground, and finally proposed humanoid walking with very high similarity to human motion. In Weon and Lee (2018), Weon et al. proposed a method for generating humanoid robot motion based on motion capture data, which corrects extracted joint trajectories based on a reprogrammed zero-moment point (ZMP) trajectory. Researchers have extensively investigated bipedal walking model-based approaches. In Graf and Röfer (2011), the three-dimensional linear inverted pendulum model (3D-LIPM) proposed is one of the most widely used simplified dynamics models for humanoid robots. The 3D-LIPM approximates the humanoid robot in the three-dimensional space to an inverted pendulum model composed of mass points and massless legs connecting the points to the support points and constrains the center of mass to move on the constrained plane. Jadidi and Hashemi (2016) proposed a closed-loop 3D-LIPM gait for RoboCup standard platform and implemented a full range of walking on the NAO robot. The 25 degrees of freedom of the NAO robot make it have excellent omnidirectional walking and full-body motion performance. The RoboCup 3D simulation team uses the NAO robot as a reference model. At the same time, this model is also widely used in robot simulation competitions at all levels at home and abroad. Astudillo et al. (2018) used the 3D-LIPM as a model for robot and ZMP to map joint angles to achieve a humanoid robot walking on a slippery platform.

As the degrees of freedom of humanoid robots increase, the complexity of systems will increase as well. Bipedal walking model-based methods will not be sufficient for the development of humanoid robot control (Fayong et al., 2014). Besides, a variety of intelligent control methods are developed, which do not require accurate modeling (MacAlpine et al., 2015; Hong and Lee, 2016; Bonyadi and Michalewicz, 2017). However, the process of adjusting motion parameters and posture based on various models is very tedious and time-consuming. When the given parameters are not reasonable, walking instability and robots moving at a low speed may occur. This shortcoming can have a significant impact on the coordination between the needs of speed, stability, and flexibility. Therefore, various intelligent algorithms are used for robot gait planning. Many researchers have applied the central pattern generator (CPG) to generate gait trajectories, but the parameter optimization of this method is a challenge (Bai et al., 2019). Zhong et al. (2017) transformed phase signal from CPG output into a trajectory signal for the legs of a six-legged robot by adjusting it. Wang et al. (2019) proposed a gait planning method based on a reactive neuromuscular controller and CPG to achieve a power-saving human-like large walk, and the controller parameters were optimized based on an optimization algorithm in the paper. Common optimization algorithms such as central force optimization (CFO) and genetic algorithm (GA) have also been successfully used for the gait planning of humanoid robots. Kumar et al. (2018) applied GA to optimize parameters for a triple-linked humanoid robot to achieve numerical simulation of energy-controlled stable walking. Huan et al. (2018a) used CFO to optimize the foot lift amplitude of a humanoid robot, which caused an efficient and stable gait. PSO is a common intelligent optimization algorithm that solves global optimization problems simply and efficiently (Kennedy and Eberhart, 1995). Huan et al. (2018b) applied PSO to optimize joint angles to achieve stable walking for a humanoid robot with 10 degrees of freedom. Mandava et al. proposed a multi-objective particle swarm optimization algorithm method for the gait optimization of humanoid robots for the trolley table model. This method uses a sliding mode controller to optimize robust tracking control and realizes the 3D simulation walking of a humanoid robot (Mandava and Vundavilli, 2018). Gülcü and Kodaz (2015) improved the performance of comprehensive learning particle swarm optimizer (CLPSO) through parallel computation and proposed PCLPSO. Most of the studies mentioned earlier are devoted to single movements such as straight, rotating, and going up and down stairs (Faraji et al., 2019). There are relatively few studies that comprehensively consider bipedal robot forward and rotation and their arbitrary motion connection transitions. Also, during the simulation process, the individual simulation platforms always add noise in the same way, resulting in similar movements of the bipedal robots. Parallel optimization algorithms can be a good solution to this problem. Muniz et al. (2016) optimized the keyframe movements of a humanoid robot (getting up, kicking, etc.) through parallelization to improve the motion performance of the robot. However, parallel algorithms suffer from low fault tolerance when dealing with distributed tasks in RoboCup3D, and it is difficult to ensure the correctness and stability of the running process.

Based on the considerations mentioned earlier, this pa*per se*lects 13 key parameters that affect speed and stability based on the gait planning method of natural ZMP trajectories and designs two evaluation functions to address the problems of humanoid robot walking. By changing the cluster structure of the PCLPSO algorithm, a parallel distributed multi-robot gait training environment is established by using the RoboCup3D simulation platform. The training environment consists of multiple computers that can operate independently. The nodes use the computer network for information transfer to achieve a common task (gait optimization for humanoid robots). The computational efficiency is improved by running in parallel in a distributed environment. A layered learning approach is used to optimize the evaluation function layer by layer. Experimental results show that the optimized humanoid robot has a faster and more stable straight gait and excellent turning ability and has less wobble when switching between straight and turning.

## Gait Parameter Selection Based on Natural Zero-Moment Point Trajectory Planning

In this paper, after setting a natural ZMP trajectory from heel to toe movement based on a 3D-LIPM in the single-leg support phase, a mass-centered trajectory equation is obtained (Graf and Röfer, 2011). In the double-leg support phase, a linear pendulum model was used to generate mass-centered trajectory equations. Equations for multistep planning of mass-centered trajectories in a unified coordinate system are also given. After planning the walking trajectory by natural ZMP-based mass-centered trajectory planning method, the key gait parameters are selected and optimized based on the experience of manual tuning.

### Multistep Trajectory Planning in a Unified Coordinate System

**Figure 1:**
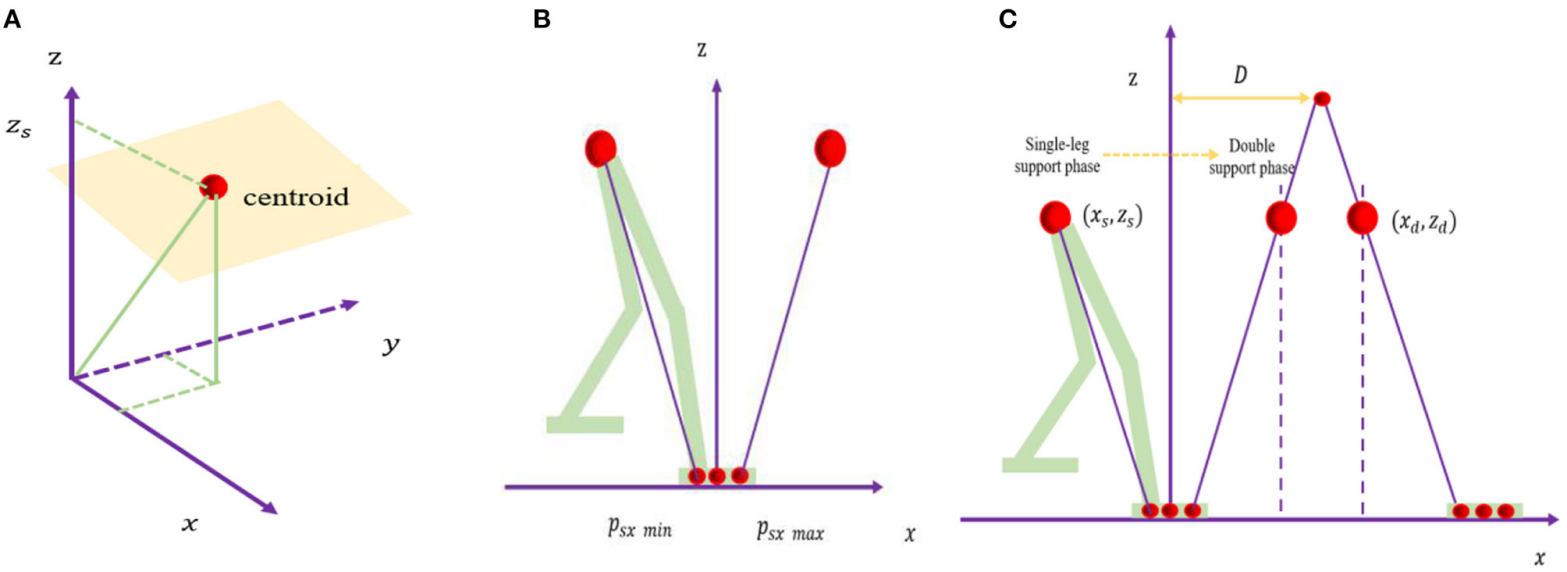
**(A)** Three-dimensional linear inverted pendulum model. **(B)** Diagram of natural zero moment point. **(C)** Linear pendulum model.

During the movement of the humanoid robot, if only the front and back and up and down movements are considered and the left and right movements are ignored, it is easy to cause the robot to lose its balance and fall. Therefore, it is necessary to extend the linear inverted pendulum to a three-dimensional environment and model the robot with a three-dimensional linear inverted pendulum. However, the process of directly analyzing and researching the multi-link structure of biped robots is often cumbersome, so this article equivalently simplified the robot to a reasonable mathematical model, which is convenient for research. Regarding the body of the robot as a mass point and the legs as massless support rods, a three-dimensional linear inverted pendulum model can be built. It does not need to know the parameters of the robot, such as the mass and the inertia of each joint, but is derived from the model for easy calculation. According to 3D-LIPM, a humanoid robot is simplified to an inverted pendulum with only a center of mass and a retractable massless pendulum, and the height of mass is assumed to remain constant, as shown in Figure 1A.

Assume that the height of the center of mass is fixed at *z*_*s*_ and the acceleration of gravity is *g*, the equation of motion between ZMP trajectory *p*_*sx*_(*t*) and the center of mass in the x-axis direction is:

(1) $$\begin{array}{l} p_{sx}\;(t)=x_{s}\;(t)-\frac{z_{s}}{g}{ẍ}_{s}\left(t\right) \end{array}$$

To make the ZMP trajectory of humanoid robot walking similar to that of human walking, this paper uses a linear equation to represent the ZMP trajectory, as shown in Figure 1B. Assuming that in the single-leg support phase, ZMP moves in the x-axis direction in the range of [*p*_*sx min*_, *p*_*sx max*_], the walking period of the single-leg support phase is *T*_*s*_; the center of the foot is the origin of a coordinate system, and the trajectory of ZMP is given by the following equation:

(2) $$\begin{array}{l} p_{sx}\;(t)=b_{1}t+b_{0} \end{array}$$

where, *t* ∈ [0, *T*_*s*_], *b*_0_ = *p*_*sx min*_, *b*_1_ = (*p*_*sx max*_ − *p*_*sx min*_)/*T*_*s*_.

By defining $w_{s}=\sqrt{\frac{z_{s}}{g}}$ by substituting Equation (1) into Equation (2) and solving a differential equation, one has:

(3) $$\begin{array}{l} x_{s}\;(t)=C_{1}e^{t/w_{s}}+C_{2}e^{-t/w_{s}}+b_{1}t+b_{0} \end{array}$$

(4) $$\begin{array}{l} {ẋ}_{s}\;(t)=C_{1}e^{t/w_{s}}/w_{s}-C_{2}e^{-t/w_{s}}/w_{s}+b_{1} \end{array}$$

If the position and velocity *x*_*s*_ (0) of the center of mass at the initial moment of the single-leg support phase and ẋ_*s*_(0) are known, then one has:

(5) $$\begin{array}{l} C_{1}=\left(\left(x_{s}\;(0)-b_{0}\right)+\left({ẋ}_{s}\;(0)-b_{1}\right)\;w_{s}\right)/2 \end{array}$$

(6) $$\begin{array}{l} C_{2}=\left(\left(x_{s}\;(0)-b_{0}\right)-\left({ẋ}_{s}\;(0)-b_{1}\right)\;w_{s}\right)/2 \end{array}$$

If the positions of the center of mass at the initial moment of single-leg support phase and the positions of the center of mass at the moment of termination *x*_*s*_(0) and *x*_*s*_(*T*_*s*_) are known, there are:

(7) $$\begin{array}{r} C_{1}=(\left(x_{s}\;(0)-b_{0}\right)\;e^{{-T}_{s}/w_{s}}-\left(x_{s}\;(T_{s})-b_{1}T_{s}-b_{0}\right)/\\ \left(e^{{-T}_{s}/w_{s}}-e^{T_{s}/w_{s}}\right) \end{array}$$

(8) $$\begin{array}{r} C_{2}=(\left(x_{s}\;(0)-b_{0}\right)\;e^{T_{s}/w_{s}}-\left(x_{s}\;(T_{s})-b_{1}T_{s}-b_{0}\right)/\\ \left(e^{T_{s}/w_{s}}-e^{{-T}_{s}/w_{s}}\right) \end{array}$$

According to Equations (2–4), the centroid trajectory planning based on natural ZMP can be realized in the single-leg support phase.

The direct application of the single-leg support phase method for walking requires the assumption that the support leg switch is instantaneous. This will cause the center of mass acceleration to jump from the maximum to the minimum. To obtain a smooth center-of-mass velocity trajectory, the legs support phase is introduced. In this paper, a linear pendulum model is used to realize natural ZMP trajectory planning of the double-leg supporting phase.

In the double-leg support phase, according to the linear pendulum model, as shown in Figure 1C, the equation of the relationship between the position of robot center of mass and acceleration is given as follows:

(9) $$\begin{array}{l} x_{d}\;(t)-D=\frac{z_{d}}{g}{ẍ}_{d}\;(t) \end{array}$$

where *z*_*d*_ < 0, *t* ∈ [0, *T*_*d*_], *T*_*d*_ is double-leg support phase walk period, and *D* is an x-axis coordinate of the fixed end of the linear pendulum.

With $w_{d}=\sqrt{\frac{-z_{d}}{g}}$, from Equation (9) can have:

(10) $$\begin{array}{l} x_{d}\;(t)=\left(x_{d}\;(0)-D\right)\cos\left(t/w_{d}\right)+{ẋ}_{d}\;(0)\;w_{d}\sin\left(t/w_{d}\right)+D \end{array}$$

(11) $$\begin{array}{l} {ẋ}_{d}\;(t)={ẋ}_{d}\;(0)\cos\left(t/w_{d}\right)-\frac{x_{d}\;(0)-D}{w_{d}}\sin\left(t/w_{d}\right) \end{array}$$

In the double-leg support phase, the starting position and speed and the ending position and the speed of the center of mass can be known, that is, *x*_*d*_ (0), ẋ_*d*_ (0), *x*_*d*_ (*T*_*d*_), and ẋ_*d*_ (*T*_*d*_) can be known.

(12) $$\begin{array}{l} D=\frac{{ẋ}_{d}{\left(0\right)}^{2}w_{d}^{2}-{ẋ}_{d}{\left(T_{d}\right)}^{2}w_{d}^{2}+x_{d}{\left(0\right)}^{2}{-x}_{d}{\left(T_{d}\right)}^{2}}{2\left(x_{d}\;(0)-x_{d}\;(T_{d})\right)} \end{array}$$

(13) $$\begin{array}{l} T_{d}=w_{d}\arccos\frac{\left(x_{d}\;(T_{d})-D\right)\left(x_{d}\;(0)-D\right)+{ẋ}_{d}\;(0)\;{ẋ}_{d}\;(T_{d})\;w_{d}^{2}}{{\left(x_{d}\;(0)-D\right)}^{2}+{ẋ}_{d}{\left(0\right)}^{2}w_{d}^{2}} \end{array}$$

A smooth center-of-mass trajectory based on natural ZMP trajectory can be achieved in the double-leg support phase according to Equations (10–13).

The methods mentioned earlier have their own coordinate systems in the single-leg support phase and double-leg support phase, which do not facilitate multistep planning calculations for footprint planning. To do so, the equations mentioned earlier need to be unified in the same coordinate system. The equation for the position and velocity of the center of the mass in multistep planning can be expressed by the following equation:

(14) $$\begin{array}{l} x\left(t\right)=\overset{n}{\underset{i=0}{\sum }}\left(x_{si}\;(t)+x_{di}\;(t)\right) \end{array}$$

(15) $$\begin{array}{l} ẋ\left(t\right)=\overset{n}{\underset{i=0}{\sum }}\left({ẋ}_{si}\;(t)+{ẋ}_{di}\;(t)\right) \end{array}$$

The robot gait realized by Equations (14, 15) yields a natural ZMP trajectory.

### Swing Leg Trajectory Planning

Cosine functions and Bessel curves can all be used to plan swing-leg trajectories (Kajita et al., 2002; Grzelczyk et al., 2016). However, the humanoid robot is divided into two phases of single-leg support phase and double-leg support phase during walking, and only when the speed and acceleration of swing-leg start and landing are zero can the humanoid robot maintain stability when switching between single-leg support and double-leg support (Tang et al., 2003). To obtain a smoother trajectory of the oscillating leg, this paper chooses the method of simple harmonic motion synthesis:

(16) $$\begin{array}{l} z_{sw}\;(t)=\{\begin{array}{c} H_{sw}\left(\frac{2}{T_{s}}t-\frac{1}{2\pi }\sin\left(\frac{4\pi }{T_{s}}t\right)\right),t\epsilon \left[0,T_{s}/2\right]\;\\ H_{sw}\left(-\frac{2}{T_{s}}t+\frac{1}{2\pi }\sin\left(\frac{4\pi }{T_{s}}t\right)+2\right),t\epsilon \left[T_{s}/2,T_{s}\right] \end{array} \end{array}$$

(17) $$\begin{array}{l} x_{sw}\;(t)=D_{sw}\left(\frac{t}{T_{s}}-\frac{1}{2\pi }\sin\left(\frac{2\pi }{T_{s}}t\right)\right) \end{array}$$

where *D*_*sw*_ and *H*_*sw*_ are the maximum height of step length and leg lift, respectively.

### Selection of Gait Parameters

There are two kinds of bipedal walking pattern generation methods. The first method uses precise knowledge of robot dynamics parameters, such as mass, centroid position, and inertia of each joint to configure walking mode. Therefore, the method mainly depends on the accuracy of the model data. In contrast, the second method uses limited knowledge of dynamics, such as the total position of the center of mass, the total angular momentum, etc. The simplified model method (3D-LIMP) used in this paper belongs to the second type. After the optimized trajectory is obtained, the robot can execute according to the corresponding trajectory, and an excellent walking gait can be obtained. By means of a gait generation method based on natural ZMP trajectory planning, humanoid robot walking is summarized in the following algorithmic steps.

**Figure 2:**
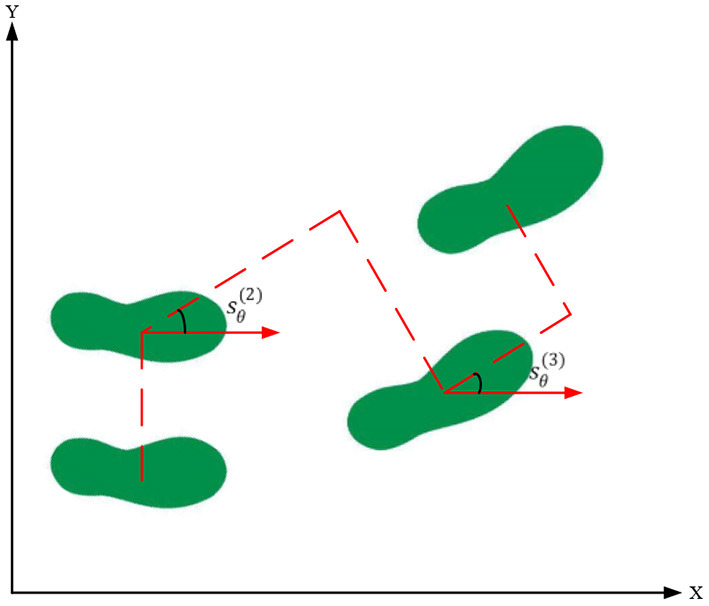
Diagram of turning parameters.

The RoboCup3D server communicates with the robot once every 20 ms. In this article, the robot is also controlled once in 20 ms. One step is eight times, so the walk period *T*_*s*_ is 0.16 s. As algorithm 1 only considers the robot walking in a straight line, for the case of steering walk. If the step length is set to zero, the robot will only walk laterally, and if the step width is set to zero, the robot will only walk in a straight line. However, in a simulation match, the flexibility of the player is enhanced by changing the direction of travel while walking quickly. To be able to walk at any angle, additional information about the direction is required. The direction of each step is specified as *s*_θ_, as shown in Figure 2.

**Algorithm 1:** 

1.Set the walking cycle *T*_*s*_ and walking parameters (*D*_*sw*_, *H*_*sw*_), initial foothold ($p_{x}^{\left(0\right)}$, $p_{y}^{\left(0\right)}$); 2.Initialization time *T* = 0, number of walking units *n* = 0; 3.Calculate the inverted pendulum equation from time *T* to *T* + *T*_*s*_ and get the equation of mass center trajectory; 4. *T* = *T* + *T*_*s*_, *n* = *n* + 1; 5. Calculate and determine the next foothold of the biped robot ($p_{x}^{\left(n\right)}$, $p_{y}^{\left(n\right)}$); 6. Give the next position of the bipedal robot; 7. Return to step 3.

Equation (18) represents the position of the nth step foothold of a humanoid robot ($p_{x}^{\left(n\right)}$,$\;p_{y}^{\left(n\right)}$)

(18) $$\begin{array}{l} \left[\begin{array}{c} p_{x}^{\left(n\right)}\\ p_{y}^{\left(n\right)} \end{array}\right]=\left[\begin{array}{c} p_{x}^{\left(n-1\right)}\\ p_{y}^{\left(n-1\right)} \end{array}\right]+\left[\begin{array}{c} \cos s_{\theta }^{\left(n\right)}-sins_{\theta }^{\left(n\right)}\\ \sin s_{\theta }^{\left(n\right)}\cos s_{\theta }^{\left(n\right)} \end{array}\right]\left[\begin{array}{c} \Delta x_{x}^{\left(n\right)}\\ -{\left(-1\right)}^{n}s_{y}^{\left(n\right)} \end{array}\right] \end{array}$$

Therefore, only important parameters such as step length, step width, and directional angle of humanoid robot need to be controlled to enable robot to walk. The gait parameters of the biped robot are as many as 40. If each parameter is optimized, it will inevitably affect the convergence speed because of the huge state space. And different gait parameters bias the focus of walking differently. According to natural ZMP-based mass-centered trajectory planning method and the previous experience of manual debugging, 13 key parameters affecting the gait of humanoid robot were selected, as shown in Table 1.

**Table 1:** Optimized gait parameters.

**Parameter**	**Description**
*FootLength*	Step size of one step (*D*_*sw*_)
*FootWidth*	Step width of one step
*FootHeight*	Maximum height of swinging leg (*H*_*sw*_)
*s*_θ_	One step turning angle
*T*_*s*_	One step time
*BodyHeight*	Body height in walking
*Thigh*	Leg length
*Hip*	Hip height
*Com*_*off*_*(x,y)*	Deviation of centroid
*Com*_max_	Offset of maximum center of mass
*Com*_*next*_	Next centroid position
*Arm(x,y)*	Amplitude of arm swing
*Zmp*_*off*_	Lateral offset of zero moment point

## Gait Optimization Based on Parallel Multigroup Particle Swarm Algorithm

Aiming at the problem that a lot of manual debugging time is required when planning the robot trajectory by directly using a simplified model, this paper proposes a machine learning method based on the PCLPSO algorithm to optimize gait parameters. First, the important parameters are extracted according to the centroid trajectory planning method based on natural ZMP. Secondly, in the optimization of walking mode, different evaluation functions are set according to the requirements of game gait mode, and the PCLPSO algorithm is used to optimize the omnidirectional walking ability of a humanoid robot.

### Parallel Comprehensive Learning Particle Swarm Optimizer Algorithm

PSO algorithm is a fast and efficient optimization algorithm (Kumar et al., 2018). Multiple individuals search for the target in the search area. It is a parallel and random optimization algorithm. Compared with other intelligent algorithms, it has a faster convergence speed and robustness. Each particle in PSO calculates the evaluation value based on the evaluation function. During the search of each particle, two extreme values are compared: the first is the optimal solution *pbest* of a particle; the other is the global optimal solution *gbest*. The speed and position updates of PSO are shown in Equations (19, 20):

(19) $$\begin{array}{l} v_{i}^{k+1}=v_{i}^{k}+c_{1}*r_{1}*\left(pbest_{i}^{k}-x_{i}^{k}\right)+c_{2}*r_{2}*\left(gbest^{k}-x_{i}^{k}\right) \end{array}$$

(20) $$\begin{array}{l} x_{i}^{k+1}=x_{i}^{k}+v_{i}^{k+1} \end{array}$$

where *i* = 1, …, *N* is the number of populations and *k* = 0, …, *N*_*iter*_ is the number of iterations; $pbest_{i}^{k}$ represents the local optimal solution found by the particle itself, whereas *gbest*^*k*^ represents the global optimal solution for all current particles; *c*_1_ and *c*_2_ are two constants greater than zero, which are used to adjust the degree of attraction of local and global optimal to the particle; *r*_1_ and *r*_2_ are both uniformly distributed random numbers in the interval [0,1], which affects the random nature of the algorithm. CLPSO is a valid variant of PSO (Liang et al., 2006). The main difference between CLPSO and PSO is that the original PSO requires the use of *pbest* and *gbest*, whereas for CLPSO, updating the location only requires *pbest*. For PSO, *pbest* only needs its own *pbest* in CLPSO update, which can come from other individuals. The speed update formula is shown as follows:

(21) $$\begin{array}{l} v_{i}^{k+1}=v_{i}^{k}+c*r*\left(pbest_{f_{i}\left(d\right)}^{k}-x_{i}^{k}\right) \end{array}$$

where *c* is the acceleration factor. *r* is also uniformly distributed random numbers in the interval [0,1]. **f**_**i**_ = [*f*_*i*_ (1), *f*_*i*_ (2), ⋯ , *f*_*i*_ (*D*)] is determined by the probability of population *P*_*c*_ of randomly selected particles *i*. $pbest_{f_{i}\left(d\right)}^{k}$ represents the pbest value of particle, which is stored in the list **f**_**i**_ of the particle *i* of the dth dimension. *P*_*c*_ is calculated, as shown in Equation (22); 0.05 and 0.45 are the optimal values of hyperparameters set by Liang et al. (2006) based on experience.

(22) $$\begin{array}{l} P_{c}=0.05+0.45*\frac{e^{\left(\frac{10\left(i-1\right)}{ps-1}\right)}}{e^{10}-1} \end{array}$$

where *ps* is the population size.

In PCLPSO, the particle population is divided into multiple groups, including a master group and several slave groups. All clusters run PCLPSO in the cluster environment at the same time. The *gbest, lbest*, and *pbest* in PCLPSO are defined as follows: *gbest* is the global optimal solution for all groups, *lbest* is the local optimal solution for populations, and *pbest* is the optimal solution for particles. Each slave group sends its *lbest* to the master group. The master group chooses the best solution from all *lbest* as *gbest* and sends *gbest* to all slaves. Each slave group randomly selects a particle to receive *gbest* to update its own *pbest*. PCLPSO algorithm updates its own *pbest* by a parallel distributed collaborative strategies. They are improving the quality of the solution and the rate of solving. Each particle in the PSO algorithm is updated by updating *pbest* and *gbest* values. The *gbest* affects the direction of population, and when it falls into a local minimum, the swarm particles tend to fall into this local minimum. PCLPSO adopts a comprehensive learning strategy, and the speed and position of the updated particles depend on all other particles. The *lbest* is selected from the *pbest* of the slave group, and the main group selects the optimal solution *gbest* from all the *lbest* of the slave group to ensure that it will not fall into a local minimum.

### Construction of a Parallel Distributed Training Environment

The particles in the group in the PCLPSO algorithm are all running on a single computer, but each football robot in a RoboCup3D match is controlled by a separate client. Therefore, the PCLPSO algorithm cannot be directly applied to the RoboCup3D simulation platform. Because all clients are connected to the RoboCup3D simulated football server, the client and server can run on multiple computers in a distributed environment. Therefore, the PCLPSO algorithm is decomposed into three algorithms by changing the cluster structure to accommodate the RoboCup3D simulation platform in this paper.

**Figure 3:**
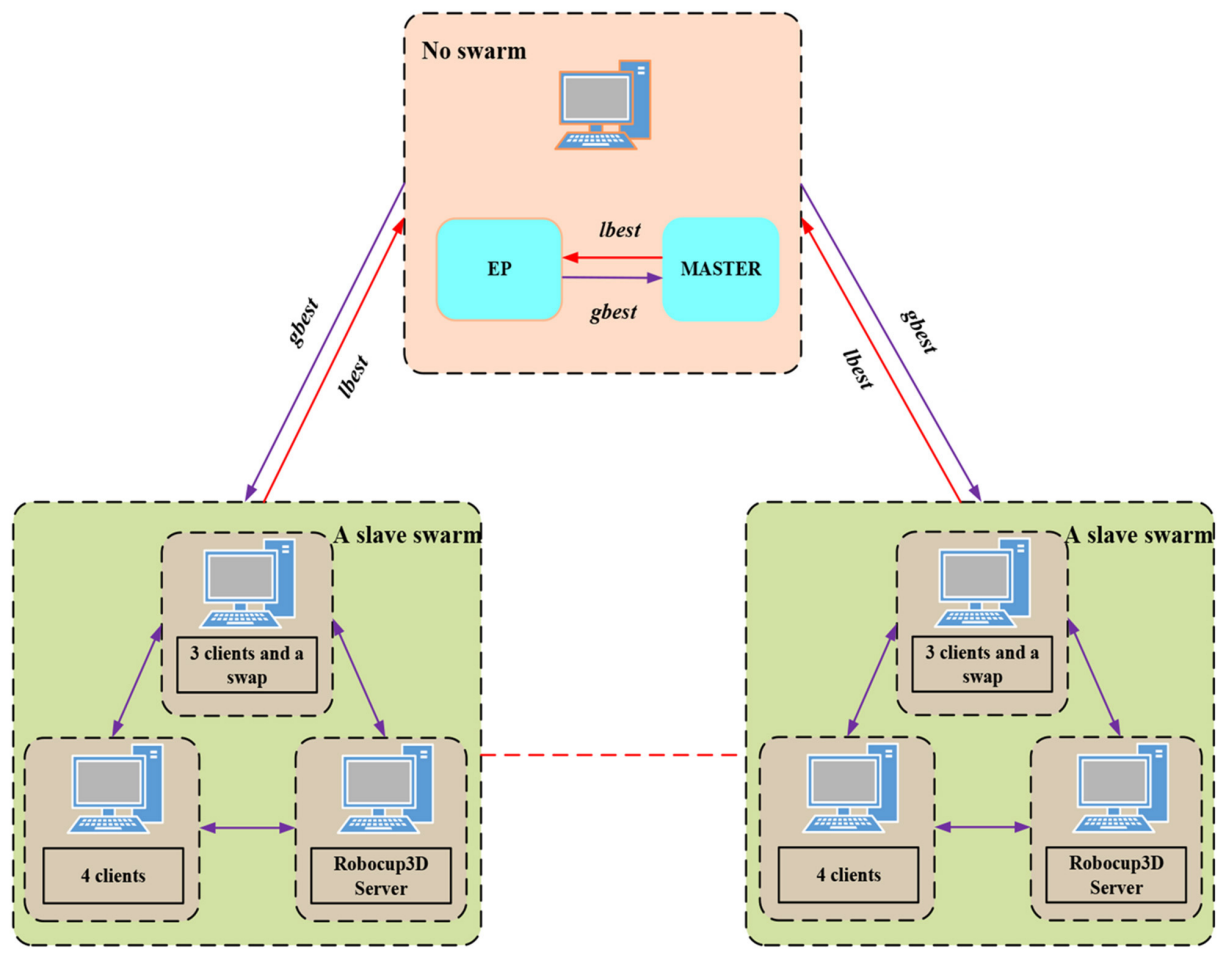
Cluster structure.

**Figure 4:**
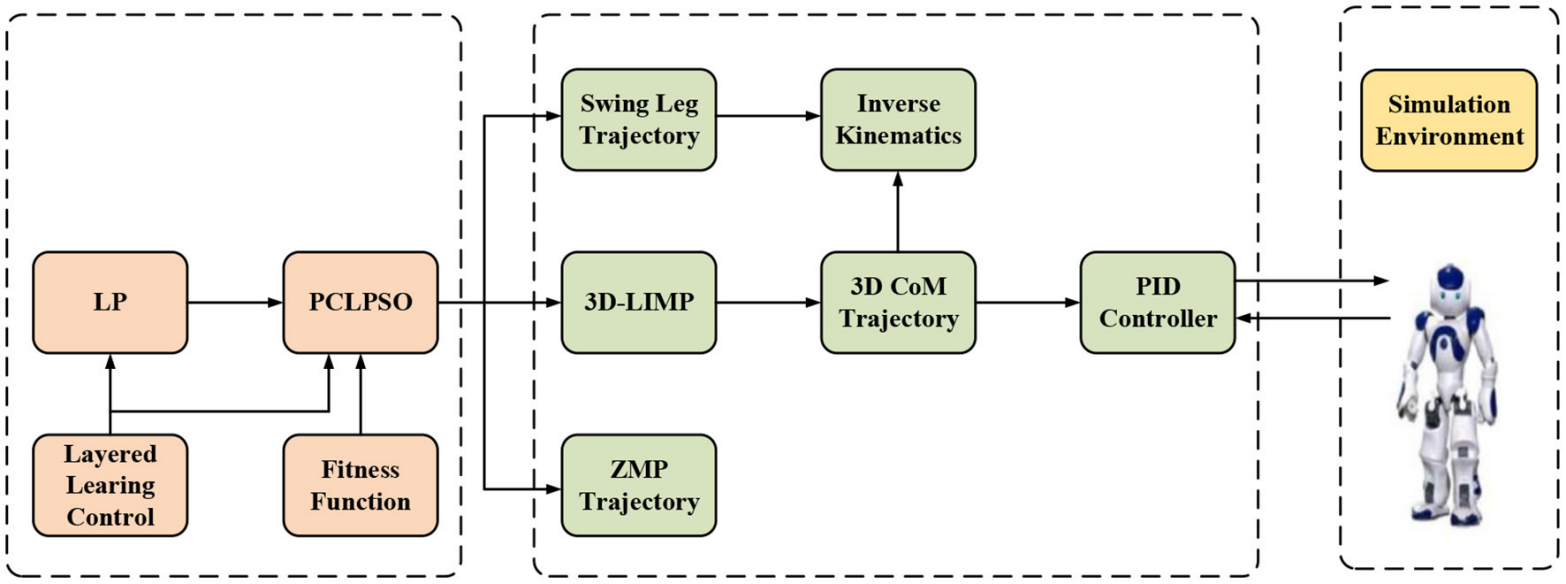
Flowchart of PCLPSO-based gait optimization.

In the new cluster, the structure is still based on the master–slave model of parallel computing; only one is a master cluster; the others are slaves, as shown in Figure 3. The gait training task of the humanoid robot is decomposed into multiple processes in the slave cluster, and a distributed training system is built to run on multiple computers. The parallel and distributed optimization framework can reduce the scale of solving gait problems. When all slaves are started and connected to the master group, and the master group sends parameters to the slave group, the communication cycle begins. Communication takes place only between the master group and slave groups; there is no communication between slave groups. In the cluster structure of this paper, the master node has no group, and its main function is to send initial parameters to the group of slave nodes (Algorithm 2, line 6), and the slave group sends its own *lbest* to the master group (Algorithm 2, line 8). The master group collects the received *lbest*, including its own *lbest*, into a pool called Elite Pool (EP). The master group finds *gbest* from the EP and eventually sends *gbest* to the slave group (Algorithm 2, lines 9, 10, and 11). The detailed algorithm of the master is shown in Algorithm 2. The slave group exchange algorithm is shown in Algorithm 3. Add an exchange program to slave group clients for exchanging data between the master client and all the clients of the slave group. In this paper, Local Pool (LP) is added to the slave group to collect all *pbest* in the group. The slave group finds *lbest* among all *pbest* and sends its own *lbest* to the master group. The master server adds them to EP, finds *gbest*, and shares it with the slave server. A robot is a member of a group during gait optimization training, a slave group has seven clients, and a client controls a humanoid robot, and eventually, multiple humanoid robots are trained in a RoboCup3D simulation court. The group client is used to calculate ZMP trajectory, COM trajectory, and swing leg trajectory when a humanoid robot is trained by the PCLPSO algorithm to walk. This part also calculates joint angle information and adaptation values for a cycle of humanoid robot walking. The detailed algorithm from the group client is shown in Algorithm 4. The gait optimization process for the client-based PCLPSO algorithm is shown in Figure 4.

**Algorithm 2:** Master algorithm

1. int *n, k, P, m* 2. double *w, c1, c2* 3. Array EP 4. Initialize the parameters *n, k, P, m, w, c1, c2* 5. Wait until all slave swarms have been launched and connected to this Master 6. The master sends the parameters to the slaves 7. **repeat** 8. Wait until all slave swarms have sent *lBest* to the master 9. The master stores the *lBests* into the EP 10. The master finds the *gBest* in the EP and empties the EP 11. The master sends the *gBest* to the slaves 16. **until** the stopping criterion is met 17. **return**

**Algorithm 3:** Switching algorithm of a slave swarm

1. int *n, k, P, m* 2. double *w, c1, c2* 3. Array LP 4. Connect to the Master until the parameters are received from the master 5. int *d* = 0 6. **repeat** 7. *d*++ 8. Store the *pBests* into the LP until all *pBests* in this slave swarm are received 9. **if** *d* mod *P*==0 **then** 10. Find the *lBest* in the LP 11. Send the *lBest* to the master 12. Wait until the *gBest* is received from the master 13. Randomly update a *lBest* with the *gBest* in the LP 14. **end if** 15. Send the LP to the all clients in this swarm 16. **until** the stopping criterion is met 17. **return**

**Algorithm 4:** Algorithm in a client of a slave swarm

1. int *n, k, P, m* 2. double *w, c1, c2* 3. Array LP 4. Connect to the swap program in a swarm 5. Wait until the parameters are received from swap program in a swarm 6. Initialize the parameters′ and velocity 7. Calculate Swing leg Trajectory and all joint angles of Robot NAO in a walk cycle 8. Humanoid robot performs a walking training and calculates the fitness value 9. Update *pBest* 10. Send the *pBest* to the swap program until the LP is received from the swap program 11. Update the LP and find the *lBest* in the LP 12. **repeat** 13. Update the velocity and position 14. Calculate Swing leg Trajectory and all joint angles of Robot NAO in a walk cycle 15. Humanoid robot performs a walking training and calculates the fitness value 16. Update *pBest* 17. Send the *pBest* to the swap program until the LP is received from the swap program 18. Update the Local Pool and find the *lBest* in the LP 19. **until** the stopping criterion is met 20. **return**

### Design of Evaluation Functions

The design of evaluation criteria is critical to achieving an excellent result (MacAlpine and Stone, 2018). In this paper, gait optimization results are judged based on the following criteria:

1. The distance the humanoid robot travels per unit time.

(23) $$\begin{array}{l} f_{dis}=a_{1}*\|\varphi_{end}-\varphi_{start}\| \end{array}$$

where φ_*start*_ and φ_*end*_ are the coordinates of start and end of the training, respectively, and *a*_1_ is the weight.

2. Whether zero force matrix is always within the supported polygon and robot walks without falling all the time. in this paper, the zmp coordinates of the walking action sequence are chosen as the basis for calculation.

(24) $$\begin{array}{l} f_{zmp}=a_{2}*\overset{N}{\underset{k=1}{\sum }}\sqrt{{\left(P_{x}\left(k\right)\right)}^{2}+{\left(P_{y}\left(k\right)\right)}^{2}} \end{array}$$

where *P*_*x*_ (*k*) and *P*_*y*_ (*k*) are the ZMP coordinates for each gait action sequence, and *a*_2_ is the weight.

3. Humanoid robot in the fast walking process torso always maintains stability; body torso does not shake. In a simulation match, the body of players is in frequent contact with each other, and it is very easy to be knocked down by the opponent if the torso is not stable. At the initial time of double-support phase, the expected com coordinates are 2 ft on the center. In this paper, torso sway is detected by comparing the two coordinates of com and the center of feet during the initial stage of the dual support phase.

(25) $$\begin{array}{l} x_{f}=c_{x}-\frac{x_{foot}R+x_{foot}L}{2} \end{array}$$

(26) $$\begin{array}{l} f_{shake}=\{\begin{array}{ll} 0 & f_{abs}\;\left(x_{f}\right)<th_{\theta }\\ c & otherwise \end{array} \end{array}$$

where *x*_*foot*_*R* and *x*_*foot*_*L* are the coordinates of initial time 2 ft of the double support phase, *th*_θ_ is the set threshold, and *f*_*shake*_ and *c* are penalty and normal numbers, respectively.

If the robot falls during training, a constant *f*_*falling*_ will be given as a penalty value. The evaluation function for speed and stability training is shown in Equation (27).

(27) $$\begin{array}{l} F_{1}=f_{dis}-f_{zmp}-f_{falling}-f_{shake} \end{array}$$

Add the lateral walking and the turning angles of the humanoid robot to the above, and the maximum distance of lateral walking for each step is limited to 0.04 m, and the maximum turning angle is 15°. In the optimization process, two different target points are set for the bipedal walking robot to train its gait. If the target point is reached, then the training of the next target point is quickly stopped. The evaluation function is as follows:

(28) $$\begin{array}{l} F_{2}=f_{dis}-f_{falling}-f_{punish}+f_{reward} \end{array}$$

where *f*_*punish*_ is the penalty for not completing the task within the specified time, and *f*_*reward*_ is the reward for completing the task.

### Layered Learning

**Figure 5:**
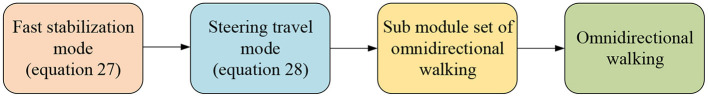
Layered learning for omnidirectional walking process.

In the RoboCup3D simulation game, players can be roughly divided into two categories. The first category is to hold the ball, avoid the defense of the opponent, intercept the ball in the shortest time, and send the ball to the goal of the opponent; the second category is to run according to the situation on the field players and walk to the designated location as soon as possible. Players without the ball often complete tasks such as running and intercepting opposing players with the ball up and down. For players to complete the tasks assigned by their superiors in the shortest possible time, the walking speed of robots is very important. The player is the defensive object of an opponent after getting the ball, so frequent collisions and turns are inevitable. Improve the stability of the robot and steering ability to avoid falling and taking advantage of collisions. The omnidirectional walking of a biped robot can be decomposed into forward walking and steering motion. This paper uses a layered learning method to optimize each decomposition action of omnidirectional walking, and the final omnidirectional walking optimization is shown in Figure 5. Each sub-module requires the optimization algorithm to be trained through the corresponding evaluation function. Use manually adjusted parameters to drive the robot to walk, and then learn to get a fast mode with speed and stability as the goal. The final optimized parameters of the fast and stable mode are used as the initial state of the steering mode, and the learning is continued with the goal of steering stability. Through hierarchical learning of the humanoid robot, two different walking parameter sets (straight and turning) can be obtained. When different walking tasks are to be completed, the parameter sets can be switched at any time, and the flexible connection transition during switching is also obtained through learning.

## Experimental Results and Analysis

This article uses a 3D-LIMP model to generate gait and opens the function of adjusting walking engine parameters to the public. OpenAI Gym acts as a bridge between optimization algorithm and environment and accepts the gait parameters computed by an optimization algorithm, performs a training session in the environment, and then provides the necessary information back to the optimization algorithm. To verify the effectiveness of the PCLPSO algorithm for gait optimization, this paper compares PCLPSO with three commonly used optimization algorithms, GA, CFO, and CMA-ES (Rongyi and Chunguang, 2018), in terms of the speed, stability, and turn capability of gait. The angle of view of players is only−120–120°. To get accurate data during training, the value of setViewCones in the server is changed from 120 to 360°. It is also necessary to change the game mode to fast mode during training so that the training is not limited by time. The mathematical properties of the four optimization algorithms of GA, CFO, CMA-ES, and PCLPSO are evolutionary algorithms. Each training uses the same population size (10) and the same number of variables (13). First, randomly produce 10 sets of parameters. The humanoid robot walks according to the 10 sets of parameters. The walking speed and stability will be different. Choose the optimal one, and then iterate the formula. Generate the next generation, and repeat the execution with 10 iterations. In this article, the coding method of the GA algorithm is standard binary coding. The number of parameters determines the length of a chromosome. When calculating the evaluation function value, the binary chromosome string should be decomposed and decoded to get the real number parameters. The specific parameters of the GA algorithm are as follows: take population size *N*_*p*_ = 40, evolutionary algebra *T* = 200, and crossover probability *P*_*c*_ = 0.7, and variation probability *P*_*m*_ = 0.05. The specific parameters of the CFO algorithm are as follows: α = 0.3,β = 0.3,γ_*start*_ = 0, γ_*stop*_ = 1, and γ = 0.1.

**Figure 6:**
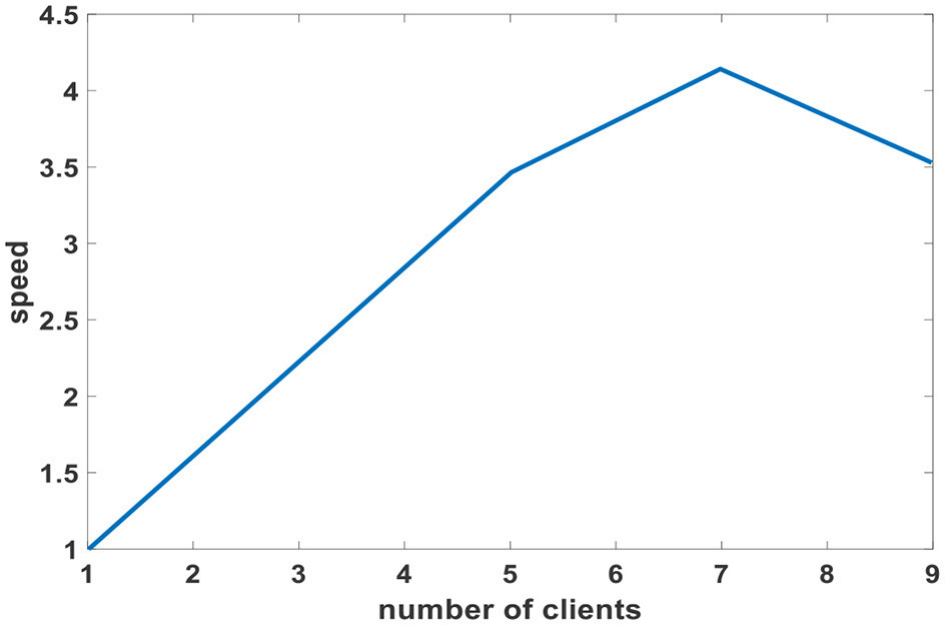
Number of clients from the group and training speed.

**Figure 7:**
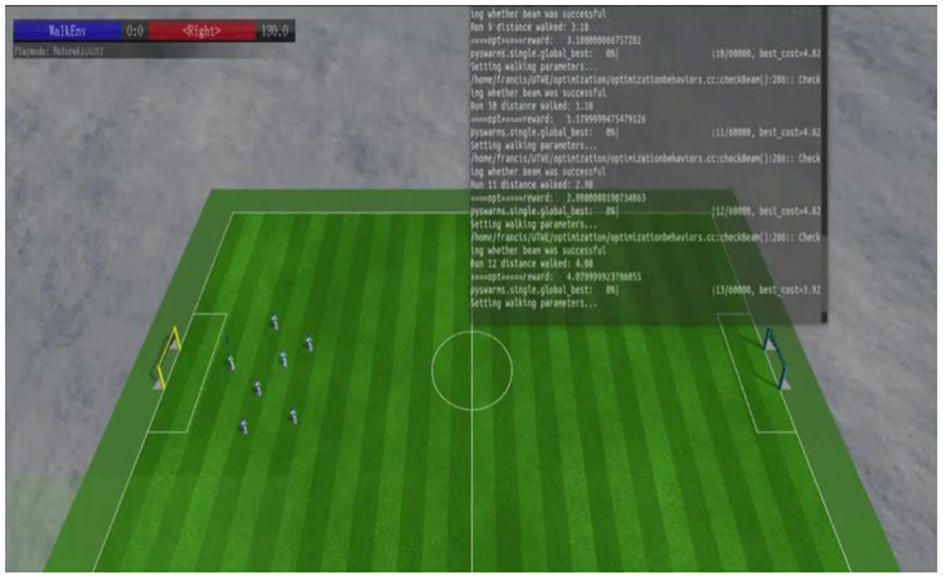
Training scenario diagram.

Firstly, Equation (27) is chosen as an evaluation function to optimize the speed and stability of the robot. The relationship between the number of clients in a single slave group and training speed is shown in Figure 6. The training speed of multiple clients is compared to a single client when it takes a single client to train to an optimal value 1. The training speed increases linearly between 1 and 7 clients. The training speed reaches its maximum when the slave group contains seven clients. So, in the following comparison experiment, the slave group structures of PCLPSO are trained by seven clients controlling seven humanoid robots. The training scenario of a humanoid robot in the RoboCup3D scenario is shown in Figure 7.

**Figure 8:**
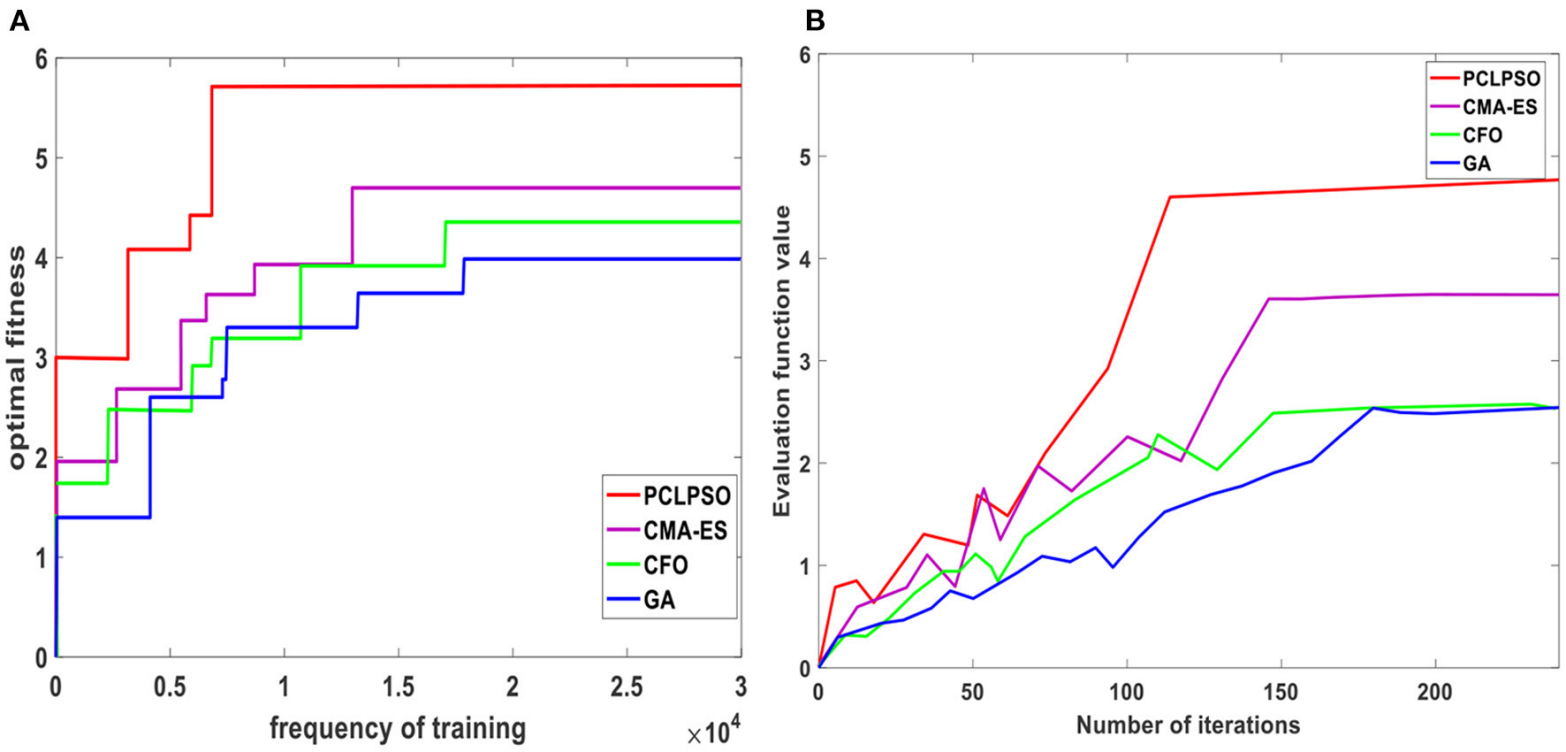
Comparison of evaluation function values of four algorithms. **(A)** Current optimal evaluation function value. **(B)** Average function value.

Figure 8A shows the current optimal fitness values of four algorithms, all of which increase with the increase in the number of training. The evaluation function of the PCLPSO algorithm reaches the optimal value after 6,500 trainings, and the optimal value is 5.86. The evaluation function of the CMA-ES algorithm reaches the optimal after 14,000 trainings, and the optimal value is 4.73. GA algorithm reaches the optimal evaluation function after 17,500 trainings, and the optimal value is 3.92. The evaluation function of the CFO algorithm reaches the optimal after 16,000 trainings, and the optimal value is 4.2. The evaluation function value is averaged every 50 times of training, as shown in Figure 8B. As the number of iterations increases, the evaluation function of all four algorithms increases steadily. One hundred twelve iterations for PCLPSO, 146 iterations for CMA-ES, 176 iterations for GA, and 148 iterations for CFO are stable. When the parallel distributed optimization framework interacts with RoboCup3D, many low-cost data samples can be obtained. Decomposition of gait problems can reduce the scale of problem-solving. Make sure to get a better solution faster.

**Figure 9:**
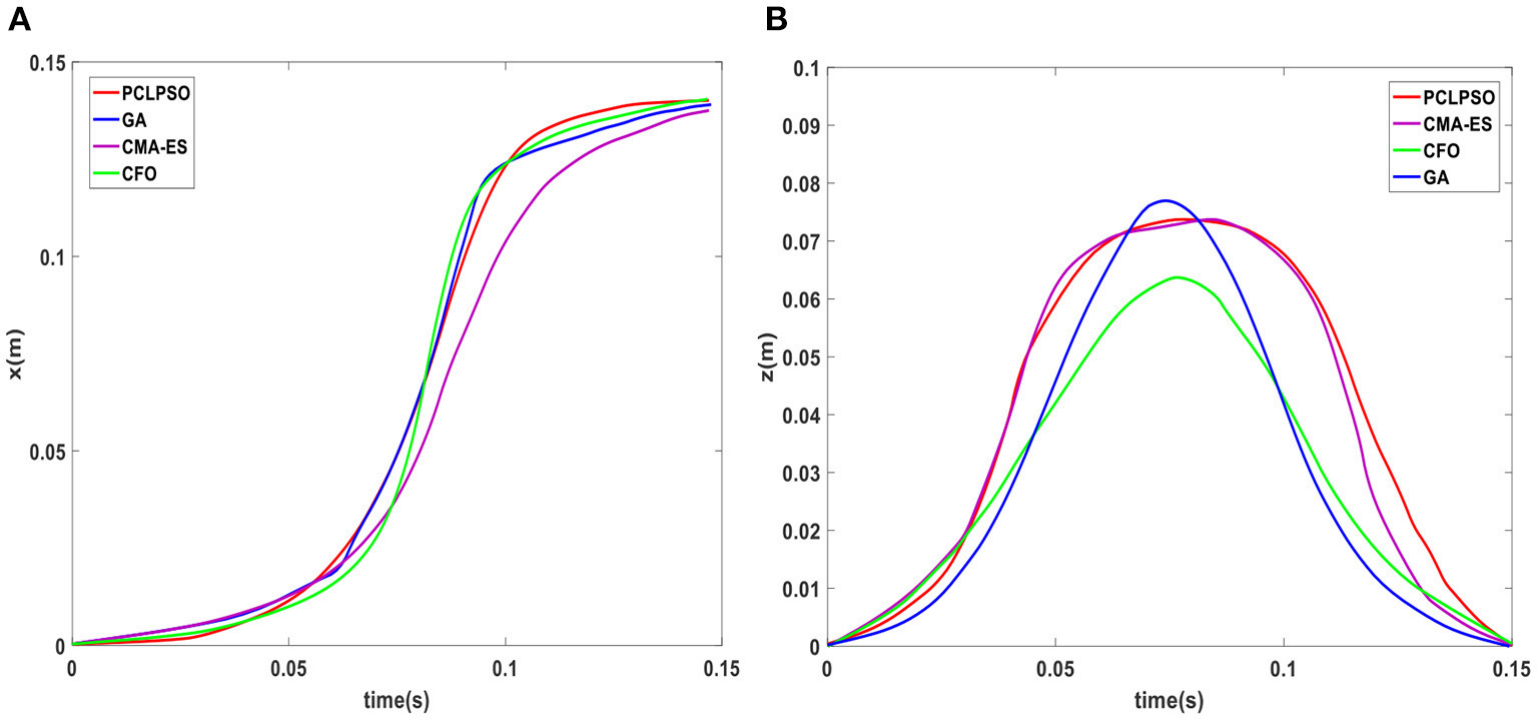
**(A)** x-axis trajectory. **(B)** z-axis trajectory.

The walking stability measured by Equation (25) shows that the *x*_*f*_ fluctuation range of the PCLPSO algorithm is−0.2 to 0.2 mm. The CMA-ES algorithm fluctuates between - 0.3 and 0.5. The *x*_*f*_ range of GA is−0.7 to 1.4 mm, and that of the CFO algorithm is−1.3 to 0.8 mm. The PCLPSO algorithm has the smallest *x*_*f*_ fluctuation range, and the humanoid robot is more stable. The optimized trajectories of swing leg x-axis and z-axis of four algorithms are shown in Figures 9A,B. At the moment of takeoff and landing, the optimized trajectory of the swing leg of the PCLPSO algorithm is parallel to the ground, which ensures stability when switching between the single-support phase and double-support phase.

**Figure 10:**
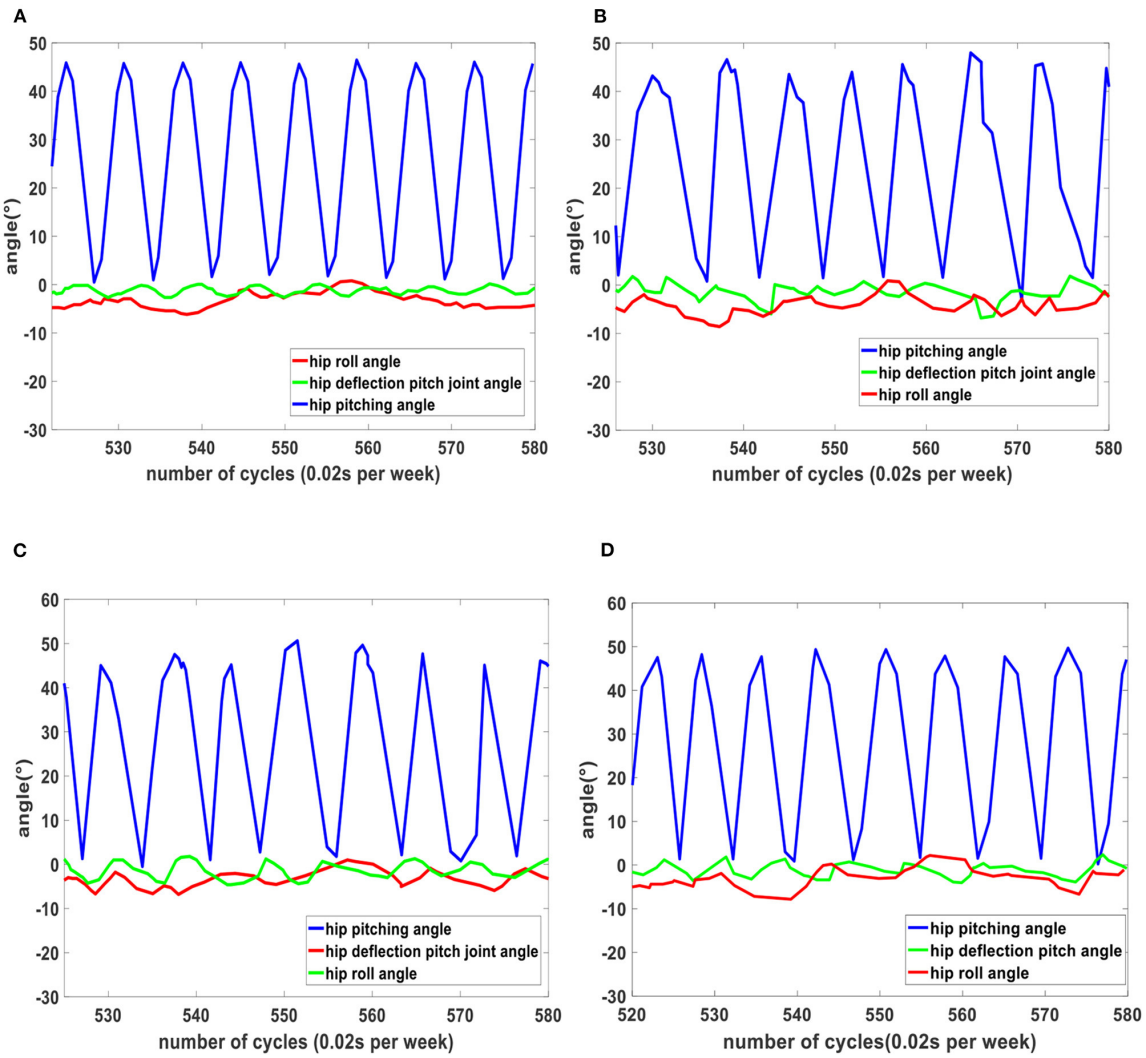
Changes in hip angle for four algorithms. **(A)** Hip angle variation of PCLPSO algorithm. **(B)** Hip angle variation of CFO algorithm. **(C)** Hip angle variation of GA algorithm. **(D)** Hip angle variation of CMA-ES algorithm.

The real-time changes of hip deflection pitch joint angle, hip transverse roll joint angle, and hip pitch joint angle for four algorithms are shown in Figure 10. The hip angle changes of the PCLPSO algorithm are very stable and better than those of the CMA-ES, CFO, and GA algorithms.

**Figure 11:**
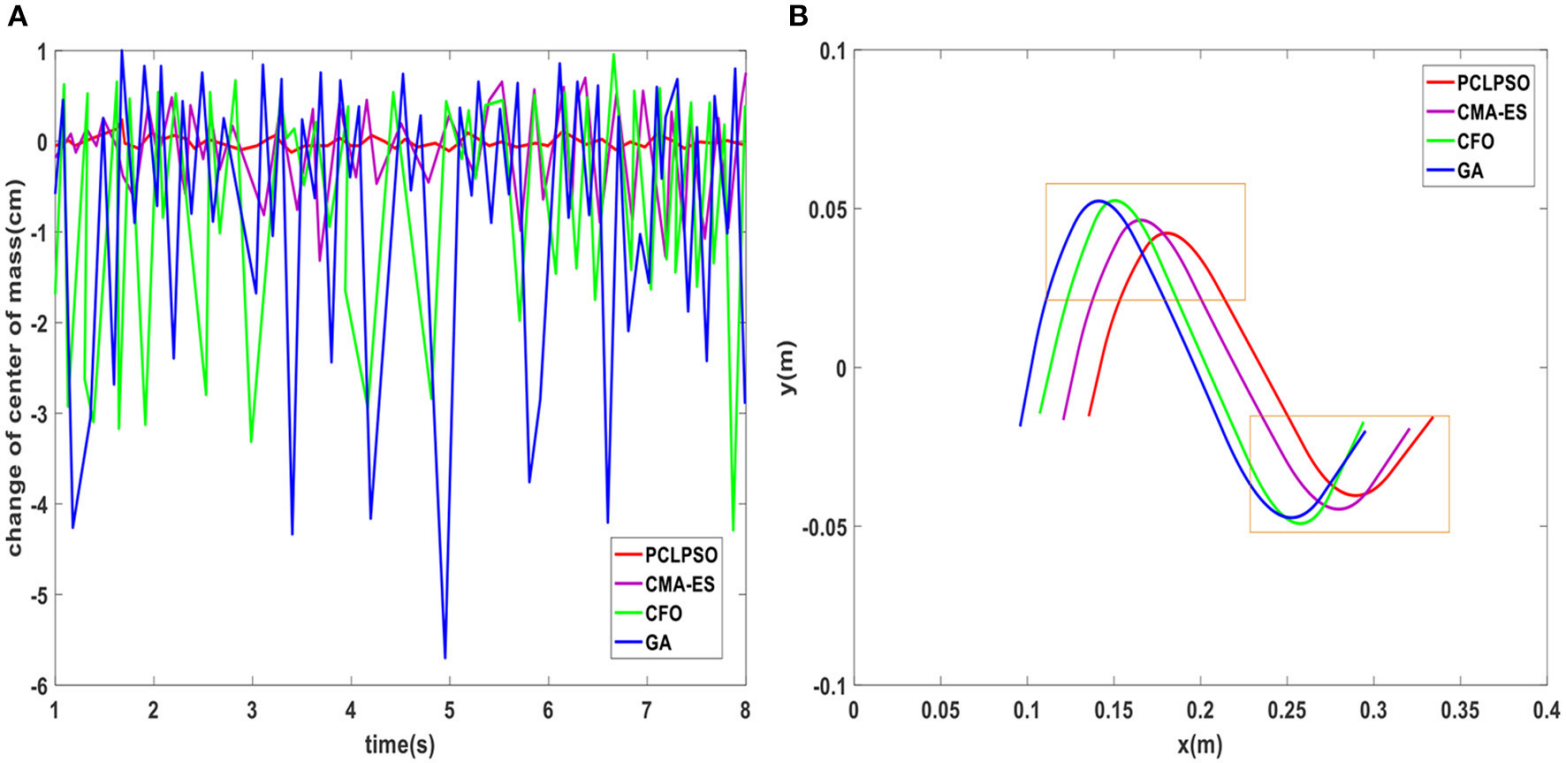
**(A)** Humanoid robot center of mass landing point *p*_*shake*_ variation. **(B)** ZMP trajectory after optimization of four algorithms.

A comparison of changes in the center of mass landing points for four methods is shown in Figure 11A. The landing point of the center of mass in the double-leg support phase of the robot using the parameters optimized by the PCLPSO algorithm is always on the center of two-legged linkage and remains stable, whereas the landing point of the robot center of mass using the parameters optimized by CFO and GA is unstable. CMA-ES algorithm optimized humanoid robot mass center fallout is more stable than the CFO and GA algorithms but inferior to the PCLPSO algorithm. ZMP trajectory after the optimization of three algorithms is shown in Figure 11B. The ZMP trajectory of the CMA-ES, GA, and CFO algorithms is close to the edge of the support polygon, whereas the whole trajectory curve of the PCLPSO algorithm moves toward the middle of the support polygon, in which case the stability margin of humanoid robot ZMP point is larger.

**Figure 12:**
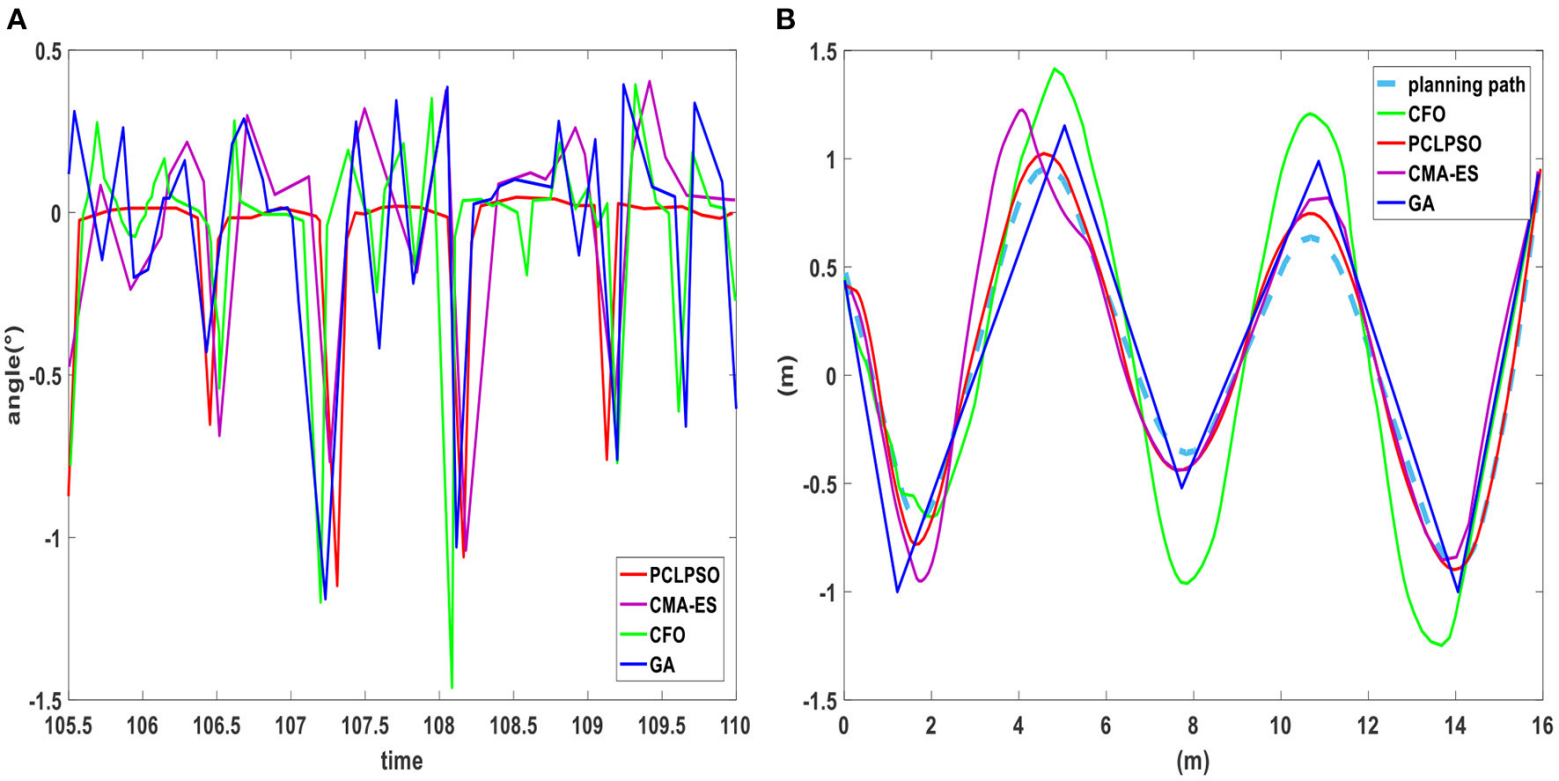
**(A)** Body rotation angle. **(B)** Turning performance test.

After the training mentioned earlier, it can be seen in humanoid robot gait optimization, and PCLPSO algorithm optimization of humanoid robot already has a fast and stable gait, but in simulation competition in a humanoid robot, the target point is constantly changing. When changing from a linear to a rotating state, the average speed decreases again when the rotation angle is small, and the humanoid robot is extremely unstable during rapid stops. In this paper, to better adapt to dynamic walking, layer learning is used to further optimize gait. The robot not only has a fast and stable gait but also has excellent steering ability. Next, the steering ability of the humanoid robot is optimized using Equation (28) as an evaluation function. Test experiments on the turning ability of humanoid robots were conducted under the SimSpark platform of RoboCup3D, and the walking path was recorded by Matlab using RoboViz observation. As shown in Figure 12A, the body rotation of four algorithms when the robot is optimized to make it turn continuously. The body rotation angle of robots using the PCLPSO algorithm with optimized parameters reaches a maximum of−1.19° during support leg switching, which shows that the robot is very stable during the switching process of walking and turning. Preplanning the walking path of the robot, the trajectory after layer learning using three algorithms is shown in Figure 12B. The GA algorithm walks a trajectory that cannot be flexible enough to maintain stability when turning. The humanoid robot optimized with the CMA-ES and CFO algorithms can turn flexibly but requires some adjustment time when turning, and the PCLPSO algorithm walks on a smooth trajectory that is almost identical to the planned path.

Let humanoid robot go straight for 15 m, test each algorithm 100 times, and take the average value. The results are shown in Table 2. The PCLPSO algorithm is used to walk with less time and faster speed under the same walking distance. The steering angle represents the angle between the body orientation of the initial position of the humanoid robot and the target point. The four algorithms are tested 100 times when the steering angle is 30, 45, 60, and 90°. The average time to reach the target point of each algorithm is shown in Table 3. The PCLPSO algorithm has the shortest average time to reach the target point at four angles.

**Table 2:** Straight speed comparison.

**Algorithm**	**Walking distance (m)**	**Average time (s)**	**Average speed (m/s)**
PCLPSO	15	16.83	0.89
CMA-ES	15	21.02	0.71
CFO	15	21.05	0.71
GA	15	25.03	0.60

**Table 3:** Steering performance test.

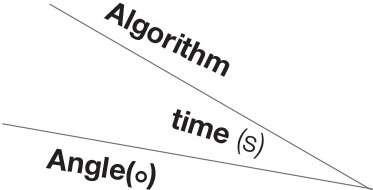	PCLPSO	CMA-ES	CFO	GA
30°	21.07	21.32	21.37	22.47
45°	21.45	22.01	22.38	22.58
60°	21.75	22.45	22.67	23.07
90°	22.40	22.73	22.85	23.39

## Conclusion

This paper has built a RoboCup3D parallel distributed multi-robot gait training environment based on PCLPSO. A layer learning approach was used to optimize the existing problems in layers. It effectively reduces the influence of similar noise of a single simulation platform and has a faster optimization speed than common optimization algorithms. The final experimental results show that the PCLPSO algorithm optimizes faster and walks more quickly and steadily. During turning motion, the PCLPSO algorithm walks with a smoother trajectory and a smaller body rotation angle when switching between straight motion and turning motion, enabling stable, and flexible turning of a humanoid robot. This algorithm can also be extended to other aspects of RoboCup3D, such as the optimization of basic movements of humanoid robots such as goal shooting.

## Data Availability Statement

The original contributions presented in the study are included in the article/supplementary materials, further inquiries can be directed to the corresponding author/s.

## Author Contributions

CT: methodology. JX: software. ZZ: funding acquisition, project administration, and supervision. FC and HG: investigation. CL: validation. All authors contributed to the article and approved the submitted version.

## Conflict of Interest

The authors declare that the research was conducted in the absence of any commercial or financial relationships that could be construed as a potential conflict of interest.
